# Draft Genome Sequence of *Idiomarina woesei* Strain W11^T^ (DSM 27808^T^) Isolated from the Andaman Sea

**DOI:** 10.1128/genomeA.00831-16

**Published:** 2016-08-11

**Authors:** Rinchen T. Lepcha, Abhijit Poddar, William B. Whitman, Subrata K. Das

**Affiliations:** a Department of Biotechnology, Institute of Life Sciences, Nalco Square, Bhubaneswar, India; b Department of Microbiology, University of Georgia, Athens, Georgia, USA

## Abstract

*Idiomarina woesei* strain W11^T^ was isolated from the Andaman Sea. Heterotrophic growth was observed at 30 to 37°C and pH 7 to 9. It grows in the presence of 0.5 to 12% (wt/vol) NaCl, and optimum growth occurred at 5 to 6% NaCl. The estimated genome is 2.4 Mb and encodes 2,305 proteins.

## GENOME ANNOUNCEMENT

*Idiomarina woesei* strain W11^T^ is a Gram-negative, short rod aerobe that belongs to the class *Gammaproteobacteria* (1). Its whole-genome sequence will help understand the halophilic lifestyle of this bacterium.

The draft genome of *Idiomarina woesei* was generated using the Illumina HiSeq 2000 platform, which generated 10,745,454 paired-end reads with a total of 1,622.6 Mb. Filtered Illumina reads were assembled using Velvet (version 1.2.07) (2). One- to 3-kb simulated paired-end reads were created from Velvet contigs using wgsim (version 0.3.0) (https://github.com/lh3/wgsim). Illumina reads were assembled with simulated read pairs using Allpaths-LG (version r46652) (3).

The genome was annotated using the JGI Microbial Genome Annotation Pipeline (4). Genes were identified using Prodigal (5), followed by manual curation using GenePRIMP (6) for finished genomes and draft genomes with <20 scaffolds (>50 kb), containing 92.9% of the genome. The final assembly was based on 1,510.0 Mb of Illumina data with 302-fold input read coverage. The predicted coding sequences (CDSs) were translated and used to search the National Center for Biotechnology Information (NCBI) nonredundant, UniProt, TIGRFam, Pfam, KEGG, COG, and InterPro databases. The tRNAscan-SE tool (7) was used to find tRNA genes, whereas rRNA genes were found by searches against models of the rRNA genes built from SILVA (8). Other noncoding RNAs, such as the RNA components of the protein secretion complex and the RNase P, were identified by searching the genome for the corresponding Rfam profiles using INFERNAL (http://infernal.janelia.org).

The draft genome assembly of *Idiomarina woesei* contained 17 contigs in 16 scaffolds, with a total genome size of 2.4 Mb and *N*_50_ contig size of 317.6 kb. The largest contig was 842.6 kb, with a G+C content of 48.0%. The draft genome sequence has 2,305 candidate protein-coding genes (CDSs), 47 tRNAs, and 8 rRNAs.

### Accession number(s).

This whole-genome shotgun project has been deposited at DDBJ/EMBL/GenBank under the accession no. LIPW00000000. The version described in this paper is the first version, LIPW01000000.
